# Feasibility Study on Shear Horizontal Surface Acoustic Wave Sensors for Engine Oil Evaluation

**DOI:** 10.3390/s20082184

**Published:** 2020-04-12

**Authors:** Saya Kobayashi, Jun Kondoh

**Affiliations:** 1 Graduate School of Integrated Science and Technology, Shizuoka University, Hamamatsu-shi 432-8561, Japan; kobayashi.saya.16@shizuoka.ac.jp; 2 Graduate School of Science and Technology, Shizuoka University, Hamamatsu-shi 432-8561, Japan

**Keywords:** shear horizontal surface acoustic wave sensor, engine oil, mechanical perturbation, electrical perturbation, particle, heating, water contained in oil

## Abstract

On site monitoring of engine oil is required. The features of a shear horizontal surface acoustic wave (SH-SAW) sensor include simultaneous detection of mechanical and electrical properties of liquids (such as viscosity, relative permittivity, and conductivity) and loaded mass on the sensor surface. In this paper, the used engine oil extracted from a motorbike was measured using the SH-SAW sensor. The degradation factors of the used engine oil were experimentally discussed. Especially, the influences of the particles in the engine oil, heating effect, and water contained in the engine oil were considered by comparing the differences between new and used engine oils. The results indicate that the influence of the water contained in the engine oil is the primary cause of the degradation of the used engine oil.

## 1. Introduction

Oil has the roles of providing cooling and normal dispersion actions, in addition to ensuring smooth operation of engine through the lubricating action. The degradation of engine oil can cause deterioration of the engine and other functions. Therefore, it is important to monitor the characteristics of the oil. For this reason, various sensors have been reported for oil monitoring. These include viscosity measurement using a Love wave [1], particle measurement in oil using a quartz crystal resonator (QCR) sensor [2], refractive index measurement using an optical fiber sensor [3], sensor utilizing the absorption of infrared light [4], complex permittivity measurement using an impedance spectroscopy [5,6,7], an ultrasonic transducer for monitoring the longitudinal wave velocity [8], water in oil [9], and free glycerol in biodiesel [10]. Also, the zero order shear horizontal plate acoustic wave [11] and lateral electric field exited resonator were applied to oil and related materials [12].

A shear horizontal surface acoustic wave (SH-SAW) sensor can detect liquid properties such as viscosity, density, relative permittivity, conductivity, and loaded mass on the SH-SAW propagating surface [13,14,15,16]. The detection mechanism of the mechanical properties and loaded mass occurs through the mechanical perturbation, and the electrical perturbations constitute the mechanism for the electrical properties [13,15,17]. The feature of the SH-SAW sensor is the simultaneous detection of the mechanical and electrical properties of a target. While the QCR sensor can measure the electrical properties of a liquid [18], the sensitivity is low due to the low electromechanical coupling factor. The Love wave sensor can be used to measure the mechanical properties of a liquid. As the Love wave is trapped in a guiding layer on a piezoelectric substrate, the sensitivity for the mechanical perturbation is higher than that of the SH-SAW sensor [19]. The sensitivity of the Love wave sensor depends on the guiding layer thickness [19]. Numerical calculations are required for the optimization of the Love wave sensor structure. This research comprises a feasibility study for applying an oil monitor based on the mechanical and electrical perturbations. Therefore, the SH-SAW sensor fabricated on 36YX-LiTaO_3_ is used. 

In our previous research, the new and used engine oils were measured using the SH-SAW sensor [16]. The used engine oil was extracted from a motorbike, with a recorded travel distance of 6000 km. The difference between the new and used oils could not be explained from the experimental results by using olive oil, which has almost the same viscosity with the new engine oil [16]. In this paper, three types of the experiments were carried out to explain the differences between the oils: (1) influences of the particles, (2) heating and oxidization, and (3) influences of water in the oil for feasibility study.

## 2. Experimental Methods

### 2.1. SH-SAW Sensor

**Figure 1:**
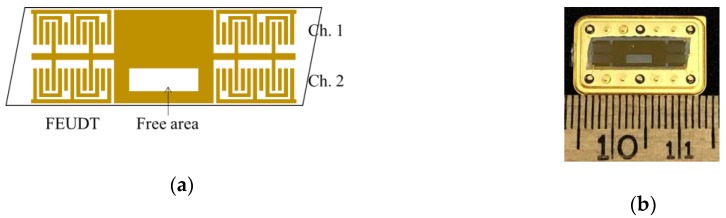
(**a**) Schematic illustration and (**b**) photograph of the shear horizontal surface acoustic wave (SH-SAW) sensor used.

A 36YX-LiTaO_3_ single crystal was selected as the substrate. Floating electrode unidirectional transducers (FEUDTs) were fabricated on the crystal surface for generating and receiving the SH-SAW [14,20]. The FEUDT was used for improving the insertion loss and phase distortion. The SH-SAW sensor consisted of a dual delay-line transversal type SH-SAW (see Figure 1). The center frequency is 51.5 MHz and the wavelength (λ) was 80 μm. The electrode materials were titanium (10 nm) and gold (200 nm). The aperture of the FEUDT was 2 mm, the number of pairs was 32, and the electrode finger width and the space between the fingers was λ/12. The center-to-center distance between FEUDTs was 11 mm. The SH-SAW propagating surface of the channel (Ch.) 1 was metallized and electrically shorted by the titanium and gold films to detect only mechanical perturbation. The thicknesses of those materials are the same as those of the FEUDT. As the SH-SAW propagating surface of the Ch.2 has the free area and a direct liquid contact with the crystal surface, the SH-SAW on the surface is influenced by mechanical and electrical perturbations. The differential signal between the Ch.1 and Ch.2 was detected for obtaining the electrical properties.

### 2.2. Measurement System

**Figure 2:**
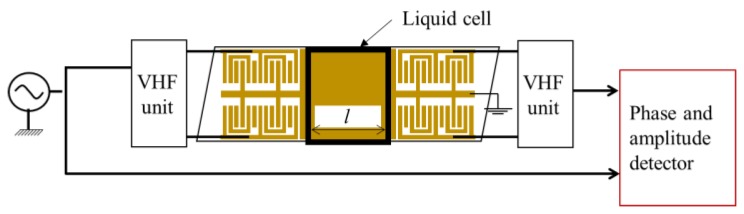
Phase and amplitude measurement system in this study. Channels 1 and 2 were selecting using the very high frequency (VHF) switching unit. The measured system was controlled by the external PC.

The experimental system used in this study is shown in Figure 2. A sinusoidal signal of 51.5 MHz from a signal generator (MG3601A, Anritsu, Japan) was divided into two signals. One of them was used for the reference signal and the other was connected to Ch.1 or Ch.2. The channels were selected using a very high frequency (VHF) switching unit (3488A, Keysight, Mansfield, TX, USA). A vector voltmeter (8508A, Keysight, Mansfield, TX, USA) was used for detecting the phase difference between the SAW sensor output signal and the reference signal, and the amplitudes of two signals. In the measurements, first, the reference liquid was injected into a liquid cell fabricated on the propagation surfaces for the calibration. Subsequently, the reference liquid was replaced by sample liquids and the changes of phase and amplitude between the reference and sample liquids were obtained for each channel. The differential signal between the Ch.1 and Ch.2 was calculated from these data using a personal computer (PC).

The measurement system shown in Figure 2 has been widely used in SAW sensor applications. However, a detailed explanation of the measurement method is not reported. In this paper, the methods employed for determining velocity and attenuation changes are summarized. A signal from a signal generator is expressed using the exponential function, which is presented in Equation (1). The signal in Equation (1) is fed into the FEUDT input and the generated SH-SAW propagates on the surface.

(1) $$F\left(t\right)=A_{0}\;e^{j\omega t}$$

Here, *A*_0_ is the amplitude and $j=\sqrt{-1}$. The signal in Equation (1) is fed to the input FEUDT and the SH-SAW generated propagates on the surface. The output signals for the reference and sample liquids loaded on the SH-SAW sensor are represented as followings:

(2) $$F_{R}=A_{0}\;e^{-\alpha_{R\;}l}\;e^{j(\omega t+\phi_{R})},$$

(3) $$F_{S}=A_{0}\;e^{-\alpha_{S}\;l}\;e^{j(\omega t+\phi_{S})}.$$

**Figure 3:**
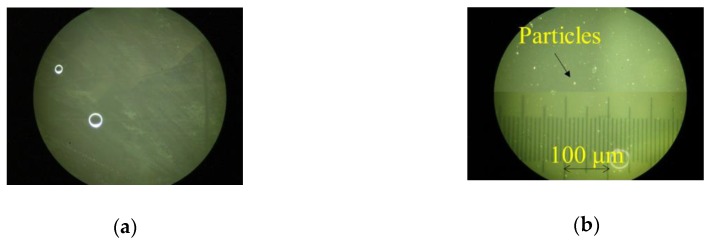
Observation results of (**a**) new and (**b**) used engine oils using the microscope.

Here, *α* is the attenuation of the SH-SAW due to the liquid loading, *l* is the interaction length between the SH-SAW and liquid, as shown in Figure 3, *ϕ* is the phase change by the liquid loaded, and the subscripts of *R* and *S* mean reference and sample, respectively. The changes in the attenuation and phase are obtained from the ratio of Equations (2) and (3).

(4) $$\frac{F_{S}}{F_{R}}=\frac{A_{0}\;e^{-\alpha_{S}\;l}\;e^{j(\omega t+\phi_{S})}\;}{A_{0}\;e^{-\alpha_{R\;}l}\;e^{j(\omega t+\phi_{R})}}=e^{-\left(\alpha_{S}-\alpha_{R}\right)\;l}\;e^{j(\phi_{S}-\phi_{R})}$$

The terms of $e^{-\left(\alpha_{S}-\alpha_{R}\right)\;l}$ and $\;e^{j(\phi_{S}-\phi_{R})}$ correspond to attenuation and phase changes, respectively. In actual measurements, the attenuation change is obtained from the amplitude of the output electrical signal.

(5) $$A_{R}=A_{0}\;e^{-\alpha_{R\;}l}$$

(6) $$A_{S}=A_{0}\;e^{-\alpha_{S}\;l}$$

Here, *A_R_* and *A_S_* are the measured amplitude for the reference and sample liquids loaded, respectively. Therefore, the attenuation change is derived as follows.

(7) $$\frac{A_{S}}{A_{R}}=\frac{A_{0}e^{-\alpha_{S}\;l}}{A_{0}\;e^{-\alpha_{R}\;l}}=e^{-\left(\alpha_{S}-\alpha_{R}\right)\;l}=e^{-∆\alpha l}\;∴∆\alpha =-\frac{\ln\left(\frac{A_{S}}{A_{R}}\right)}{l}$$

Since the attenuation change in the perturbation theory is normalized by the wave number *k*, Equation (7) is modified as

(8) $$\frac{∆\alpha }{k}=-\frac{\ln\left(\frac{A_{S}}{A_{R}}\right)}{kl}.$$

When a reference liquid is loaded on the SH-SAW sensor, whose propagation surfaces are metallized and electrically shorted, we can assume that *A_S_* equals to *A_R_*. Then, Equation (8) becomes zero. Therefore, the perturbation equation for the Newtonian fluid in Equation (A2) is rewritten as the following:

(9) $$\frac{∆\alpha }{k}=9.30\times 10^{-9}\sqrt{{\rho_{\ell }}^{\prime }\eta^{\prime }f}\;$$

The velocity change of the SH-SAW is obtained from the phase change in Equation (10).

(10) $$∆\phi =\phi_{S}-\phi_{R}=\frac{\omega l}{V+∆V}-\frac{\omega l}{V}\approx -\frac{∆V\phi }{V}\;∴\frac{∆V}{V}=-\frac{∆\phi }{\phi }$$

### 2.3. Samples

In this study, two engine oils were prepared: new engine oil (Yamaha 4 stroke motor oil, SAE 10W-40) and used engine oil. The used engine oil was extracted from a scooter (JBH-SA39J, Yamaha Motor Co., Ltd., Iwata, Japan), that had travelled 6000 km. Photographs of the engine oils are shown in Figure 12 in Reference [16]. Observation results of the engine oils with a microscope are shown in Figure 3. As particles exist in the used engine oil, it is important to discuss their influence of the particles. Although the kinds of particles have not been analyzed, iron (Sigma-Aldrich Japan, Tokyo, Japan, ϕ5–9 μm) and carbon (Fuji Film Wako Pure Chemical Co., Osaka, Japan, ϕ45 μm) powders were used in this study. The reference liquids used in this study were olive oil (Fuji Film Wako Pure Chemical Co., Osaka, Japan) or the new engine oil. The relative permittivity, conductivity, and viscosity of the olive oil were 3.1 and 0.0 S/m, and 90.0 mPas, respectively. The conductivity was measured using a conventional conductivity meter. The relative permittivity and conductivity of the olive oil was used for calculating the electromechanical coupling factor in Equations (A5) and (A6). In this study, three types of the measurements were carried out: 1) influence of the particles of iron and carbon, 2) influence of the heating, and 3) influence of the water contained in the oil. The particles were observed in the used engine oil, the influences of particles were experimentally discussed. The operating temperature in an engine is high, so the influence of the heating must be discussed. When the temperature of an engine decreases, moisture is contained in the engine oil owing to dew condensation. In this study, distilled water was mixed with the new engine oil to confirm the influence of water in the oil.

## 3. Results and Discussion

### 3.1. Measurements of New and Used Engine Oil

**Figure 4:**
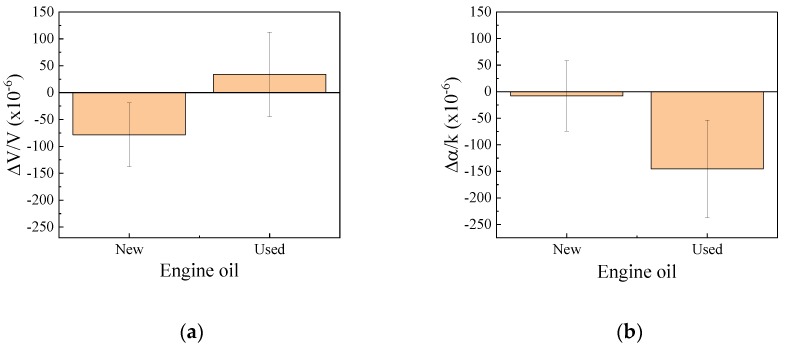
Measured results of new and used engine oils obtained from Ch.1. The reference liquid was the olive oil. (**a**) Velocity change and (**b**) attenuation change.

The results obtained from the differential signal between Ch.1 and Ch.2 are shown in Figure 13 in Reference [16] (also see Figure 7a,b and Figure 14). The results obtained from Ch.1 are shown in Figure 4. The reference liquid was olive oil. The results indicate that the viscosity of the new engine oil was lower than that of olive oil. However, the viscosity of the used engine oil cannot be explained from Equations (A1) and (A2). However, the velocity change shows an increase of the viscosity, the attenuation change shows the decrease of the viscosity. Therefore, we concluded that the used engine oil is not the Newtonian fluid.

### 3.2. Influence of the Particles in Engine Oil

**Figure 5:**
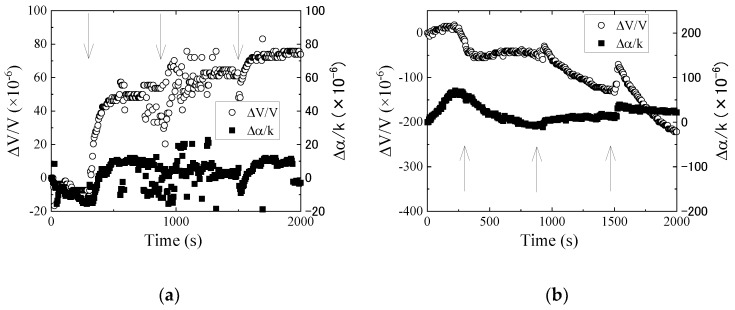
Measured results of (**a**) iron and (**b**) carbon powders in new engine oil obtained from Ch.1. The arrow indicates the addition time of the particles of 0.5 mg.

The iron and carbon particles were injected into the new engine oil. The results for the iron and carbon powders obtained from Ch.1 are shown in Figure 5a,b, respectively. The arrows in the figure indicate the addition time of the powders weighting 0.5 mg. In Figure 5a, the velocity change increases, while the attenuation change increases initially and then becomes constant. As depicted from Equations (A3) and (A4) for mass loading, the velocity change decreases, and the attenuation change is zero. The obtained results do not agree with the perturbation theory. We assumed that the increase of the attenuation change (at the first injection of the iron powders) was due to the viscosity change. The penetration depth of the SH-vibration depends on the liquid density and viscosity, and increases with an increase in viscosity [13]. As the viscosity of the engine oil was almost the same as the olive oil, the viscous penetration depth was approximately 700 nm. The powder diameter was larger than the depth, and a part of the particle existed in the penetration depth. As the SH-SAW was influenced by the change in viscosity, the attenuation change increased. The value of the attenuation change was subtracted from the velocity change to eliminate the influence of viscosity on the velocity change. However, the velocity change increased. Equations (A3) and (A4) were derived when a thin and uniform layer was loaded on the SH-SAW propagation surface. In this case, the oil exists between the particles, and the influence of oil was not considered. The increase of the velocity change was explained by considering the effective density, which was determined from the liquid and iron densities [16]. For Figure 5b, the velocity change decreases, and the attenuation change slightly increases with increasing the amount of the carbon powders. As the density of the carbon is smaller than it of the iron, the sedimentation rate of the carbon powder is less than that of the iron powder. Therefore, the velocity change did not saturate at the second and third powder injections.

**Figure 6:**
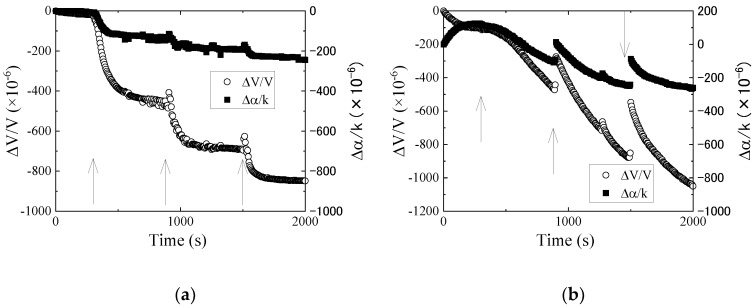
Measured results of (**a**) iron and (**b**) carbon powders in new engine oil obtained from the differential signal between Ch.1 and Ch.2. The arrow indicates the addition time of the particles of 0.5 mg.

**Figure 7:**
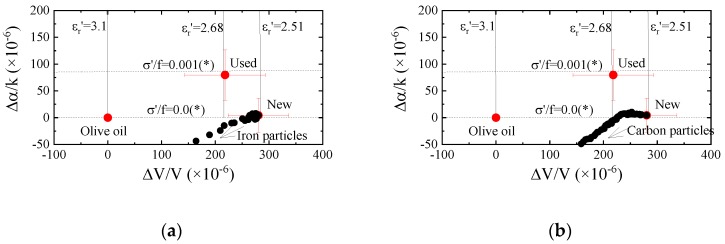
Evaluation of the electrical property changes of (**a**) iron and (**b**) carbon powders in new engine oil using the relative permittivity–conductivity chart. The arrow shows the direction of time. The results of olive oil and new and used engine oils are also plotted in the figure. (*: 1 × 10^−8^ (S/m)/Hz).

Figure 6 shows the results obtained from the differential signal between Ch.1 and Ch.2. The observed tendencies for the iron and carbon powders were almost the same. The velocity and attenuation changes decrease with the increases in the amount of powders. The velocity and attenuation changes for iron powders are saturated after each injection event. However, the changes for the carbon powder are not saturated. The behaviors of the particles are the same with Figure 5. As the reference liquid in Figure 6 was the new engine oil, the reference was numerically converted from the new engine oil to the olive oil for evaluating the electrical property changes. The results are shown in Figure 7. In the figure, the results of the new and used engine oils are also plotted. The figure indicates that the relative permittivity and conductivity of the used engine oil increased compared with the new engine oil. The relative permittivity increases with increased iron or carbon powder content. However, an increase of the conductivity was not observed. The powders in the used engine oil were observed, as shown in Figure 3b. However, the number of the particles in the used engine oil was less than the number of the particles used during the measurements. Therefore, the influences of the particles were not main causes behind the difference between the new and used engine oils. The negative changes in the attenuation could not be explained from the perturbation theory. It is necessary to reconsider the reason and clarify the negative attenuation change.

### 3.3. Influence of the Heating

**Figure 8:**
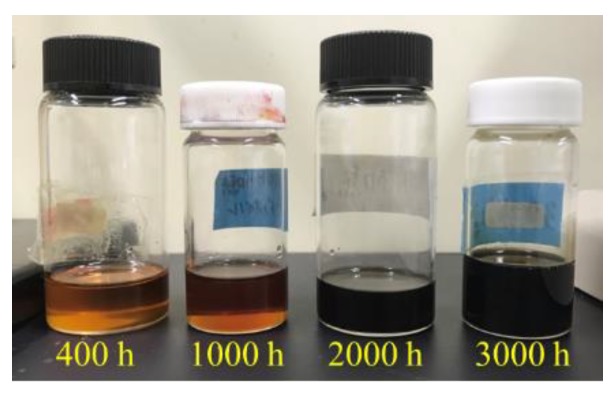
Photos of the heated engine oils. The lids of the vessels were open during the heating.

**Figure 9:**
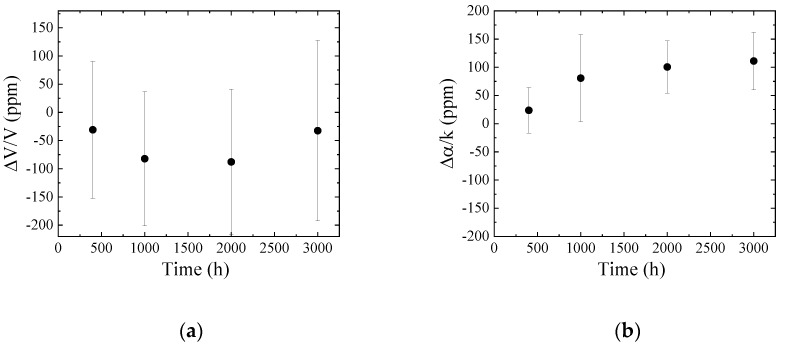
(**a**) Velocity and (**b**) attenuation changes obtained from Ch.1 as a function of heated time.

The operating temperature of the engine is high, hence, engine oil is influenced by temperature. The new engine oil was injected into a vessel and heated from 400 to 3000 h at 100 °C. To observe the influence of oxidization, the lids of the vessels were not closed. A photograph of the samples after heating is shown in Figure 8. The color depends on the heated time and changes due to the oxidization. The color after 3000 h of heating is similar to the used engine oil (see Reference [16] Figure 12). The measured results obtained from Ch.1 are shown in Figure 9. The reference liquid was the new engine oil. The mechanical properties of the liquid are influenced by the heating. When the velocity and attenuation changes of the reference liquid is assumed to be zero, the following relation is derived from Equations (A1) and (A2).

(11) $$\frac{∆\alpha }{k}=-\frac{∆V}{V}$$

**Figure 10:**
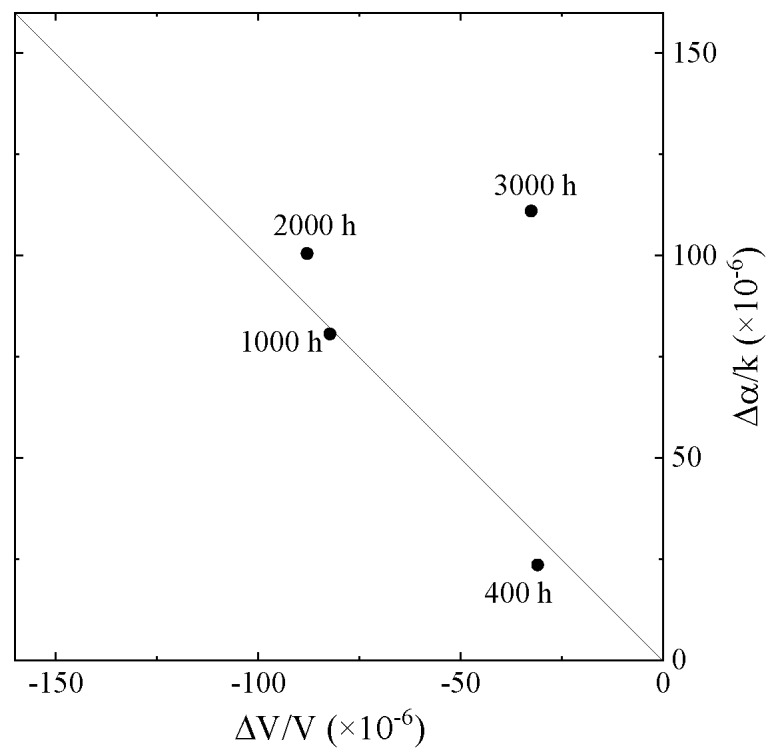
Evaluation of the SH-SAW sensor responses in Figure 9. The solid line shows the Newtonian fluid.

The average values in Figure 9 were plotted in Figure 10. As the results of 400 and 1000 h heated are on the line, those liquids are categorized as Newtonian fluid. Whereas, the sample liquids with 2000 and 3000 h heating are the non-Newtonian fluid. Compared to our previous results, those liquids can be regarded as viscoelastic fluid [14]. Therefore, the heating of the engine oil can be used to perform evaluation based on the mechanical perturbation. However, the tendencies of the measured results do not agree with Figure 4. The observed difference between the new and used engine oils in Figure 4 could not be explained by the heating effect.

**Figure 11:**
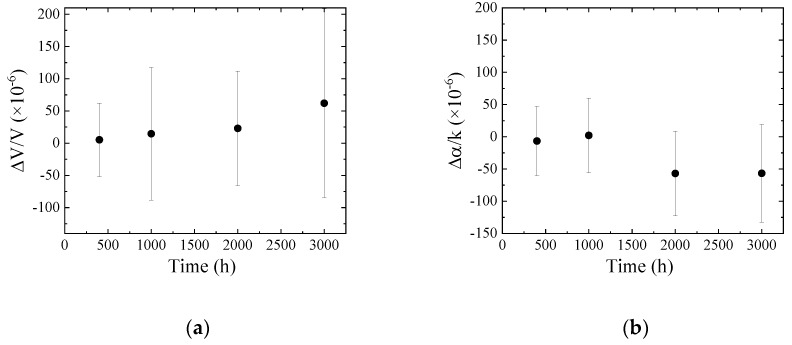
(**a**) Velocity and (**b**) attenuation changes obtained from the differential signal between Ch.1 and Ch.2 as a function of heated time.

The results obtained from the differential signals between Ch.1 and Ch.2 are shown in Figure 11. From the results, it can be observed that the change in electrical property due to the oil heating are small. The tendencies of the velocity and attenuation changes do not agree with the difference between the new and used engine oils. The electrical perturbation is not suitable to evaluate the electrical properties of the heated oil.

### 3.4. Influence of the Water Contained in the Oil

**Figure 12:**
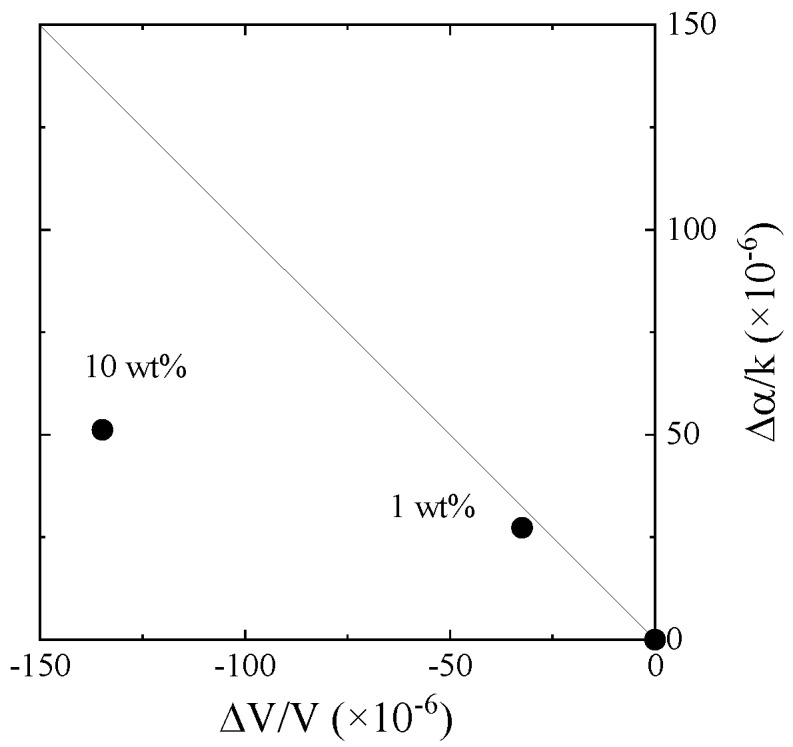
Evaluation of the SH-SAW sensor responses obtained from Ch.1 for the water contained in engine oil. The solid line shows the Newtonian fluid.

**Figure 13:**
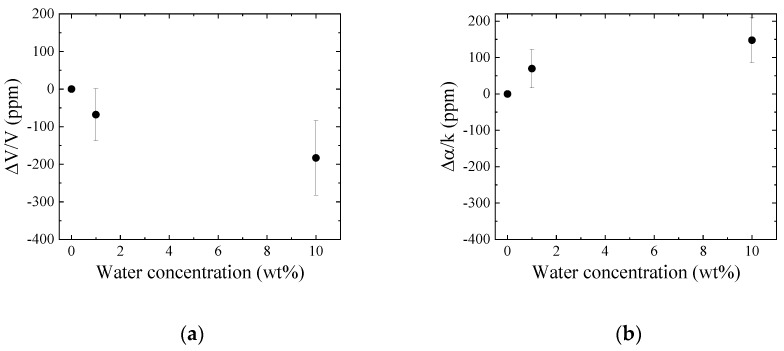
(**a**) Velocity and (**b**) attenuation changes for the water contained in engine oil obtained from the differential signal between Ch.1 and Ch.2.

**Figure 14:**
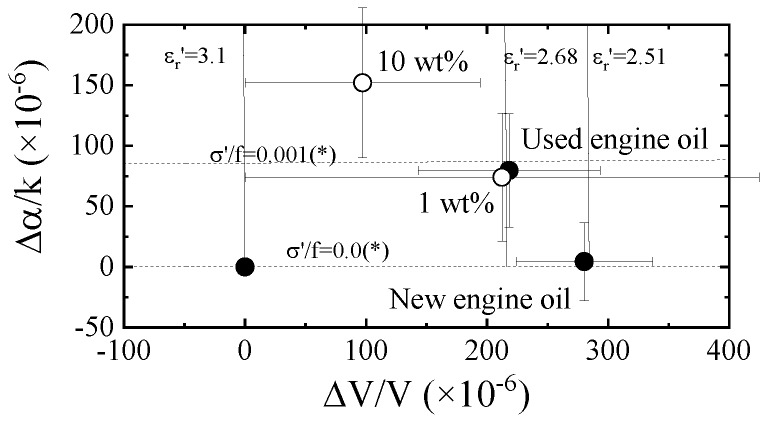
Evaluation of the SH-SAW sensor responses in Figure 13 using the relative permittivity–conductivity chart. The results of the olive oil and new and used engine oils are also plotted in the figure. (*: 1 × 10^−8^ (S/m)/Hz)

Distilled water was ultrasonically mixed with the new engine oil and the influence of the water in the engine oil was experimentally considered. The measured results from Ch.1 are plotted on the velocity change–attenuation change plane (see Figure 12). The non-Newtonian properties of the oil containing water are different after the heating effect. The results from the differential signal between Ch.1 and Ch.2 are shown in Figure 13. The velocity and attenuation changes increased with the increase in water concentration. The obtained tendencies agree with the difference between the new and used engine oils. The obtained results are plotted on the chart, as shown in Figure 14. The result of 1 wt.% water contained in the new engine oil agrees with the used engine oil. The result indicates that the primary cause of engine oil degradation is due to the influence of water. The Karl Fischer titration test [21] is used to measure the water concentration in oil. However, the real time monitoring of oil quality based on the Karl Fischer titration test is impossible, but the same is possible using the SH-SAW sensor. The wireless and passive monitoring is possible using a SAW sensor [22]. Therefore, the SH-SAW sensor is suitable for oil quality monitoring on site.

## 4. Conclusions

In this study, the differences between new and used engine oils were experimentally discussed using the SH-SAW sensor fabricated on the 36YX-LiTaO_3_. Three types of experiments were carried. The results of the 1 wt.% water contained in the new engine oil agreed with the used engine oil when the SH-SAW sensor was used for detecting the electrical properties. Therefore, the SH-SAW sensor for detecting the electrical properties could be applied for engine oil quality monitoring. The SH-SAW sensor can detect mechanical properties of liquids. When the engine oil was heated or the water was mixed to the engine oil, mechanical property changes were observed and the engine oil model changed from the Newtonian to the non-Newtonian fluid. However, the differences between new and used engine oils were not clear. The sensitivity of the SH-SAW sensor fabricated on 36YX-LiTaO_3_ for viscosity was not high. When the SH-SAW was used on the quartz substrate, the highly sensitive detection of viscosity was possible. However, the sensitivity of the SH-SAW sensor on the quartz towards the electrical properties was low. The simultaneous use of SH-SAW sensors fabricated on quartz and 36YX-LiTaO_3_ was the optimal method for ensuring highly sensitive detection. The temperature characteristics depends on the piezoelectric crystals used. The temperature correction was performed by detecting the differential signals of two SAW sensors fabricated on the same piezoelectric crystal. It is necessary to derive a new method to correct the temperature of the SAW sensor fabricated on different piezoelectric crystals. This will be addressed in our future works. It is necessary to establish a method for reducing the variations in measured values, as the error bars of the measured values in this study were large. In this study, the frequency of the SH-SAW sensor was fixed at 51.5 MHz. As the sensor sensitivity depends on the frequency, the selection of optimum frequency is also an important research subject. We must develop a compact and highly accurate measurement system for practical applications.

## Figures and Tables

**Figure A1:**
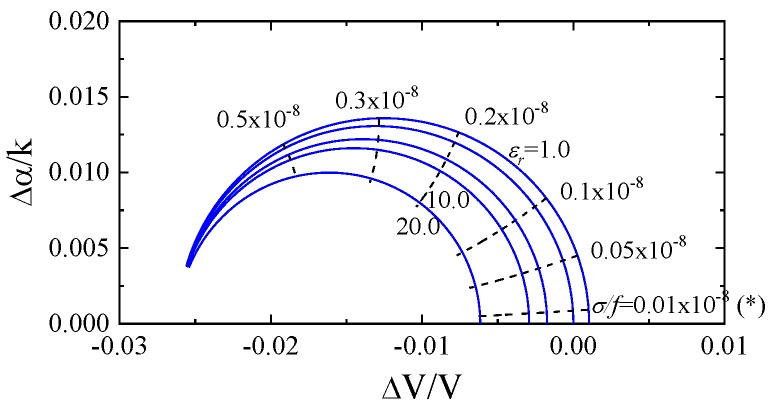
The relative permittivity–conductivity chart derived from Equations (A5) and (A6). (*: 1 × 10^−8^ (S/m)/Hz)

## Appendix A

The perturbation equations based on the Auld’s method are useful to discuss and evaluate the experimental results. A detailed description of the perturbation equations has been published [13,15]. In this paper, the equations for the three cases are described. The influences of the Newtonian fluid and mass loading are derived based on the mechanical perturbation. When the SH-SAW sensor is fabricated on the 36YX-LiTaO_3_, the constants in the equations are obtained using the material constants of the crystal.

For Newtonian fluid:

(A1) $$\frac{∆V}{V}=-9.30\times 10^{-9}\sqrt{f}\left(\sqrt{{\rho_{\ell }}^{\prime }{\eta_{\ell }}^{\prime }}-\sqrt{\rho_{\ell }\eta_{\ell }}\right),$$

(A2) $$\frac{∆\alpha }{k}=9.30\times 10^{-9}\sqrt{f}\left(\sqrt{{\rho_{\ell }}^{\prime }{\eta_{\ell }}^{\prime }}+\sqrt{\rho_{\ell }\eta_{\ell }}\right).$$

For mass loading:

(A3) $$\frac{∆V}{V}=-3.30\times 10^{-8}\left(\rho^{\prime }-\frac{\mu^{\prime }}{V^{2}}\right)hf,$$

(A4) $$\frac{∆\alpha }{k}=0.$$

Here $\frac{∆V}{V}$ and $\frac{∆\alpha }{k}$ are the velocity change and the attenuation change normalized by the wave number *k*, respectively, *f* is the frequency of the used SH-SAW sensor, *V* is the unperturbed phase velocity, $\rho_{\ell }$ and η are the density and viscosity of the liquid, respectively, ρ, μ, and *h* are the density, shear modulus, and thickness of the loaded film on the SH-SAW sensor, respectively, and the symbol of ′ denotes sample parameter.

The equations for the electrical perturbation are followings.

(A5) $$\frac{∆V}{V}=-\frac{K_{S}{}^{2}}{2}\frac{{\left(\frac{\sigma^{\prime }}{\omega }\right)}^{2}+\varepsilon_{0}\left({\varepsilon_{r}}^{\prime }-\varepsilon_{r}\right)\left(\varepsilon_{r}^{\prime }\varepsilon_{0}+\varepsilon_{P}^{T}\right)}{{\left(\frac{\sigma^{\prime }}{\omega }\right)}^{2}+{\left(\varepsilon_{r}^{\prime }\varepsilon_{0}+\varepsilon_{P}^{T}\right)}^{2}},$$

(A6) $$\frac{∆\alpha }{k}=\frac{K_{S}{}^{2}}{2}\frac{\left(\frac{\sigma^{\prime }}{\omega }\right)\left(\varepsilon_{r}\varepsilon_{0}+\varepsilon_{P}^{T}\right)}{{\left(\frac{\sigma^{\prime }}{\omega }\right)}^{2}+{\left(\varepsilon_{r}^{\prime }\varepsilon_{0}+\varepsilon_{P}^{T}\right)}^{2}}.$$

Here, *K*_s_^2^ is the electromechanical coupling factor of the piezoelectric substrate used, ω is the angular frequency of the SH-SAW sensor used, $\varepsilon_{P}^{T}$ is the effective permittivity of the piezoelectric substrate used, which is obtained from $\varepsilon_{P}^{T}=\sqrt{\varepsilon_{11}^{T}\varepsilon_{33}^{T}-{\left(\varepsilon_{31}^{T}\right)}^{2}}$, $\varepsilon_{11}^{T}$,$\;\varepsilon_{33}^{T}$, and $\varepsilon_{31}^{T}$ are the permittivity of the used piezoelectric substrate at the constant stress, respectively, $\varepsilon_{r}$ and σ are the relative permittivity and conductivity of a liquid, respectively, and $\varepsilon_{0}$ is the dielectric constant of the free space. The coupling factor depends on the reference liquid. As an olive oil was used for the reference liquid in this paper, the coupling factor of 0.05226 was calculated using the numerical calculation method based on the Campbell and Jones method [13,23,24]. The unknown parameters in Equations (A5) and (A6) are the relative permittivity and conductivity of the sample liquid. The special chart, which is called the relative permittivity–conductivity chart, was proposed as shown in Figure A1 [13]. The solid and dashed lines show the relative permittivity and the conductivity normalized by the frequency, respectively. The chart was used to evaluate the obtained results.
